# Exopolysaccharide Characterization of *Rhizobium favelukesii* LPU83 and Its Role in the Symbiosis With Alfalfa

**DOI:** 10.3389/fpls.2021.642576

**Published:** 2021-02-10

**Authors:** Lucas G. Castellani, Abril Luchetti, Juliet F. Nilsson, Julieta Pérez-Giménez, Caren Wegener, Andreas Schlüter, Alfred Pühler, Antonio Lagares, Susana Brom, Mariano Pistorio, Karsten Niehaus, Gonzalo A. Torres Tejerizo

**Affiliations:** ^1^Instituto de Biotecnología y Biología Molecular (IBBM), CCT-La Plata, CONICET, Departamento de Ciencias Biológicas, Facultad de Ciencias Exactas, Universidad Nacional de La Plata, La Plata, Argentina; ^2^CeBiTec, Bielefeld University, Bielefeld, Germany; ^3^Programa de Ingeniería Genómica, Centro de Ciencias Genómicas, Universidad Nacional Autónoma de México, Cuernavaca, Mexico

**Keywords:** rhizobia, exopolysaccharide, alfalfa, symbiosis, nitrogen fixation

## Abstract

One of the greatest inputs of available nitrogen into the biosphere occurs through the biological N_2_-fixation to ammonium as result of the symbiosis between rhizobia and leguminous plants. These interactions allow increased crop yields on nitrogen-poor soils. Exopolysaccharides (EPS) are key components for the establishment of an effective symbiosis between alfalfa and *Ensifer meliloti*, as bacteria that lack EPS are unable to infect the host plants. *Rhizobium favelukesii* LPU83 is an acid-tolerant rhizobia strain capable of nodulating alfalfa but inefficient to fix nitrogen. Aiming to identify the molecular determinants that allow *R. favelukesii* to infect plants, we studied its EPS biosynthesis. LPU83 produces an EPS I identical to the one present in *E. meliloti*, but the organization of the genes involved in its synthesis is different. The main gene cluster needed for the synthesis of EPS I in *E. meliloti*, is split into three different sections in *R. favelukesii*, which probably arose by a recent event of horizontal gene transfer. A *R. favelukesii* strain devoided of all the genes needed for the synthesis of EPS I is still able to infect and nodulate alfalfa, suggesting that attention should be directed to other molecules involved in the development of the symbiosis.

## Introduction

Alfalfa (*Medicago sativa*), is a widely cultivated legume used worldwide for feeding cattle mainly due to its high nutritional value, but also because its cultivation does not cause a high soil erosion (Li et al., 2011; Li and Brummer, 2012). This legume establishes a highly specific symbiosis with rhizobia, in which the model symbiont is *Ensifer* (*Sinorhizobium*) *meliloti*. This symbiosis allows the biological reduction of atmospheric N_2_ to plant-usable forms of nitrogen (Roy et al., 2020). The interaction between rhizobia and leguminous plants is a complex process, and needs the production of specific compounds from both organisms. A successful symbiosis requires a sophisticated exchange of signals between bacteria and plant (Oldroyd, 2013). This communication involves molecules of the plant, as flavonoids present in exudates or proteins such as lectins, and molecules produced by rhizobia: Nod factors or surface polysaccharides (Niehaus and Becker, 1998). The plant secretes a set of different flavonoids for which rhizobia have a specific receptor, the NodD protein (Peck et al., 2006). The recognition of flavonoids induces expression of Nod factors (NFs) in bacteria (Peters et al., 1986; Peters and Long, 1988), which are responsible for root hair curling and induction of cell division in the root cortex (Oldroyd et al., 2011). Bacteria proliferate within the infection thread and achieve invasion and colonization of the root interior (Jones et al., 2007).

Rhizobia can produce different types of surface polysaccharides relevant for the symbiosis establishment, such as exopolysaccharides (EPS), lipopolysaccharides (LPS), capsular polysaccharides (KPS), and cyclic β-glucans (CG) (Niehaus and Becker, 1998; Fraysse et al., 2003). The surface polysaccharides are required for a successful invasion (Fraysse et al., 2003; Kawaharada et al., 2015). Rhizobial mutants defective in the production of distinct polysaccharides show deficiencies in symbiosis establishment (Diebold and Noel, 1989; Hotter and Scott, 1991; Cheng and Walker, 1998; Fraysse et al., 2003; Hozbor et al., 2004). As examples, in *E. meliloti* 2011, a mutation of *exoB* (which encodes an UDP-glucose epimerase) leads to non-infected nodules on alfalfa (Buendia et al., 1991) and, also in *E. meliloti* 2011, mutants of *exoY* (encoding an undecaprenyl-phosphate galactose phosphotransferase) are not able to elongate infection threads (Reuber and Walker, 1993; Jones, 2012). Nevertheless, each symbiotic relationship has to be carefully evaluated, as it has been reported that a mutant of *Sinorhizobium fredii* HH103 that lacks EPS, shows an increase in the competitive capacity and forms N_2_-fixing nodules on *Vigna unguiculata* (Rodriguez-Navarro et al., 2014).

The relevance of each polysaccharide is specific for each rhizobia-leguminous plant interaction (Niehaus and Becker, 1998). Different strains of *E. meliloti* can produce more than one symbiotically active polysaccharide (Table 1). *E. meliloti* 1021 (henceforth, *Eme*1021) produces an exopolysaccharide called EPS I or succinoglycan, which is necessary for infection initiation and extension of the infection thread (Cheng and Walker, 1998). *E. meliloti* Rm41 (*Eme*Rm41) produces succinoglycan and KPS active forms, also required for infection initiation (Pellock et al., 2000). An *exoB* mutant of *Eme*Rm41 (*Eme*AK631) is defective in EPS I production but still produces KPS and shows a similar symbiotic phenotype as its parental strain, *Eme*Rm41 (Pellock et al., 2000). A derivative of *Eme*1021 with a mutation in *expR101*, acquires the capacity to produce EPS II, which differs in composition to that of EPS I. This strain showed a less effective invasion compared to the strain producing succinoglycan, but still showed induction of effective nodules (Pellock et al., 2000). Although the different surface polysaccharides can partially restore the nodulation capacity of mutants defective in EPS production, efficiency in the different steps of the nodulation process vary depending on the structure of the polysaccharide produced. Succinoglycan consists of octasaccharide repeating units of one galactose and seven glucose residues, but the repeating units can be associated with additional acetyl, pyruvyl, and succinyl groups. Cheng and Walker (1998) described that the acetyl modification is not critical for the symbiotic function of succinoglycan in *E. meliloti*, but the lack of this modification reduces the efficiency of infection thread formation. At the same time, they visualized that a deficiency in succinylation is associated with the formation of aborted infection threads. Microarrays assays of *Medicago truncatula* showed that succinylation of EPS changes the transcriptomic response during infection, especially the lack of succinoglycan enhances the transcription of plant defense genes (Jones and Walker, 2008; Jones et al., 2008). EPS can be produced in a high-molecular-weight (HMW) and a low-molecular-weight (LMW), and succinylation of EPS is necessary for the cleavage that generates the LMW (York and Walker, 1998). A mutant in *exoH* of *Eme*1021, which produces EPS I of HMW without the succinyl modification, is not able to extend infection threads and cannot perform a fruitful symbiosis (Cheng and Walker, 1998). It was recently confirmed that the succinyl modification is the essential feature, rather than the production of EPS I of LMW (Mendis et al., 2016).

**TABLE 1:** Relevant characteristics of different *E. meliloti* strains with different polysaccharides [Data obtained from Pellock et al. (2000)].

Name	Genotype	EPS I	EPS II	KPS	Efficiency in infection thread initiation	Efficiency in infection thread extension	Nodulation on alfalfa
*E. meliloti* 1021 / 2011	Wild-type	+	−	−	100%	100%	+
*E. meliloti* Rm41	Wild-type	+	−	+	100%	100%	+
*E. meliloti* AK631 (*exoB*)	*Eme*Rm41 *exoB*631	−	−	+	80%	30%	+
*E. meliloti* 9000	*Eme*1021 *expR*101 *exoY*210::Tn*5*	−	+	−	50%	20%	+
*E. meliloti* 7210	*Eme*1021 *exoY*210::Tn*5*	−	−	−	10%	0%	−

Alfalfa can also be nodulated by other rhizobia, such as the acid tolerant *Rhizobium favelukesii* (Torres Tejerizo et al., 2016). This bacterium has the ability to nodulate different *Medicago* species, *Phaseolus vulgaris* and *Leucaena leucocephala*, but this interaction leads to inefficient nitrogen fixation nodules (Eardly et al., 1985; Wegener et al., 2001). The nodules developed in alfalfa by *R. favelukesii* are white, small and contain fewer bacteroids than those developed by *E. meliloti* (Wegener et al., 2001). Nevertheless, *R. favelukesii* is very competitive for the nodulation of alfalfa in acid soils (Segundo et al., 1999). During the symbiosis between *M. sativa* and *R. favelukesii* LPU83, it was shown that this bacterium does not require sulfated forms of the NFs (Torres Tejerizo et al., 2011). This is a remarkable difference in comparison with the symbiosis of alfalfa and *E. meliloti*, which absolutely needs sulfated NFs to nodulate (Schultze et al., 1995). Moreover, in alfalfa nodules developed by *E. meliloti*, only one bacteroid is found within the peribacteroidal membrane (Vasse et al., 1990), while in *R. favelukesii* it was shown that up to six bacteroids were found within a single peribacteroidal membrane, separated by matrix material (Wegener et al., 2001). Up to now, nodulation of alfalfa by *R. favelukesii* has shown differences during nodule development, nitrogen fixation and requirement of decorations on the NFs. In this work, we aimed to analyze another of the molecules necessary for infection: the EPS, which are involved in the development of the symbiosis between alfalfa and *E. meliloti*.

## Materials and Methods

### Bacterial Strains and Plasmids

The strains and plasmids used in this work are listed in Supplementary Table 5. *Escherichia coli* was grown on Luria–Bertani (LB) (Miller, 1972) medium at 37°C. *Rhizobium* and *Ensifer* strains were grown on Tryptone–Yeast extract (TY) (Beringer, 1974),Yeast extract–Mannitol (YEM) (Vincent, 1970), or Vincent Minimal Medium (VMM) (Vincent, 1970) at 28°C. For solid media 15 g of agar per liter of medium were added. When needed, Congo Red was added at 0.25% (w/v) and Calcofluor was added at 0.02% (w/v). The final concentration of antibiotics used was (in μg ml^–1^): gentamicin (Gm) 10, kanamycin (Km) 25, and tetracycline (Tc) 10 for *E. coli*. For *Rhizobium* and *Ensifer*: streptomycin (Sm) 400, nalidixic acid (Nal) 20, neomycin (Nm) 60, rifampicin (Rif) 100, spectinomycin (Sp) 100, Gm 30, and Tc 5.

### EPS and Total Proteins Quantification

*Ensifer meliloti* and *R. favelukesii* were cultivated in YEM medium. Cultures were centrifuged 45 min at 10,000 × *g*. The EPS in the supernatant was precipitated with three volumes of ethanol overnight at −20°C and stored until processed. Then, the samples were centrifuged for 45 min at 10,000 × *g* and the pellets were resuspended in water. The quantification was performed by the anthrone method (Loewus, 1952). Amounts of EPS were normalized per mg of total proteins, which was determined by Bradford assay with Coomassie brilliant blue (Bradford, 1976).

### EPS Composition and Structure

Bacteria were grown in shaker flasks containing 200 ml of VMM at 30°C for 7 days. The precipitation and purification of EPS were done according to Müller et al. (1988). For monosaccharide analysis, the EPS was hydrolysed in 2 M trifluoroacetic acid for 2 h at 120°C in sealed glass vials. The hydrolysate was subsequently dried using a rotary evaporator. Remaining amounts of trifluoracetic acid were removed by the addition of isopropanol and dried again using the rotary evaporator. This treatment was carried out twice. Samples were dissolved in water and the sugars were analyzed by high-performance anion-exchange chromatography with pulsed amperometric detection on a Carbo Pac PA1 column (250 by 4 mm; Dionex, Sunnyvale, Calif.) and isocratic elution (1 ml/min) with 16 mM NaOH.

The EPS was analyzed by proton nuclear magnetic resonance (^1^H-NMR) spectroscopy. For ^1^H-NMR analysis, purified EPS was dissolved in deuterium oxide (99.7%), lyophilized, redissolved, and again lyophilized. Finally, 10 mg/ml of EPS was dissolved in deuterium oxide (99.99%), and sonicated for 5 min at room temperature. Spectra were recorded at 600 MHz and 80°C (Bruker Avance III–600).

### Bacterial Matings

Bacterial matings were performed as described by Simon et al. (1983). The visualization of plasmids in the transconjugants (plasmid profiles) was evaluated on Eckhardt-gels (Eckhardt, 1978) as modified by Hynes and McGregor (1990).

### DNA Manipulation and Genetic Constructs

Total DNA and plasmid preparations, restriction-enzyme analysis, cloning procedures, and *E. coli* transformation were performed according to previously established techniques (Sambrook et al., 1989). PCR amplification was carried out with recombinant *Taq* DNA polymerase or Pfu DNA polymerase as specified by the manufacturers. Primers are listed in Supplementary Table 6.

#### Plasmid Constructions and Mutagenesis

The deletion of the *exo* cluster located in the plasmid, yielding strain LPU83 Δplasmid, was generated as follows. Firstly, a fragment of 414 bp of the *exoV* gene was amplified with *Taq* polymerase and primers *exoV-BamHI-del-LEFT/exoV-XbaI-del-RIGHT* and cloned into the commercial vector pCR 2.1-TOPO (Invitrogen), obtaining pTOPO-*exoV* (4365 bp). Plasmid pHP45 Sp allows the release of the Sp resistance gene as blunt *SmaI* fragment of 2066 bp. This *SmaI* fragment of pHP45 Sp was introduced into the *EcoRV* site of pTOPO-*exoV*, selecting the construction with Km^*r*^ Sp^*r*^ Amp^*r*^ (pTOPO-*exoV*-Sp, 6431 bp). In parallel, a fragment of 382 bp of the *exoP* gene was amplified with *taq* polymerase and primers *exoP-BamHI-del-LEFT/exoP-XbaI-del-RIGHT* and cloned into the commercial vector pCR 2.1-TOPO, obtaining pTOPO-*exoPp* (4313 bp). Digestion of pTOPO-*exoPp* with *EcoRI* allows the release of the *exoPp* fragment (400 bp). The *EcoRI* fragment of pTOPO-*exoPp* was cloned into the *EcoRI* site of pK18mobSacB. The vector with the desired orientation was called pK18mobSacB-*exoPp* (6121 bp). This construction was digested with *NheI* and *SalI*, and ligated with the *SpeI*/*XhoI* product of TOPO-*exoV*-Sp (2576 bp), which contains both the *exoV* fragment and the Sp resistance gene, generating pK18mobSacB *exoV*-Sp-*exoPp* (8505 bp). To introduce the pK18mobSacB-*exoV*-Sp-*exoPp* into *R. favelukesii* LPU83, firstly pK18mobSacB-*exoV*-Sp-*exoPp* was transformed in *E. coli* S17-1 and then matings were carried out. Double recombinants were selected as Nm^*s*^, and Sp^*r*^. To corroborate the insertion, PCRs were carried out with primer *Sm-Sp* and *L_exoV-out-LEFT* (682 bp) and *Sm-Sp* and *L_exoP-out-RIGHT* (626 bp).

For the construction of strain LPU83 Δchromo, fragments of *exoZ* and *exoP* genes were amplified (185 and 210 bp, respectively), using *Phusion* polymerase (Thermo Scientific) and primers *exoZ_Fw_cro83_Eco/exoZ_Rv_cro83_Sma* and *exoP_Fw_cro83_Bam/exoP_Rv_cro83_Hin*, respectively. The fragments were cloned into the *SmaI* site of pK18mob. pK18mob-*exoPc* (4027 bp) was digested with *BamHI* and *HindIII*, and the released fragment (216 bp) was cloned into pK18mobSacB, obtaining pK18mobSacB-*exoPc* (5907 bp). Then, an *EcoRI*/*SmaI* fragment from pK18mob-*exoZ* (193 bp) containing the *exoZ* fragment was cloned into the *EcoRI*/*SmaI* sites of pK18mobSacB-*exoPc*, obtaining pK18mobSacB-*exoZ-exoPc* (6082 bp). To add the Tc resistance gene, the *SmaI* fragment of pHP45 Tc (2160 bp) containing the Tc resistance gene was cloned into the *SmaI* site of pK18mobSacB-*exoZ-exoPc*, selecting the Km^*r*^ Tc^*r*^ vector, called pK18mobSacB-*exoZ*-Tc-*exoPc*. This vector was introduced by conjugation into *R. favelukesii* LPU83. Double recombinants were selected as Km^*s*^, and Tc^*r*^. To corroborate the insertion, PCRs were carried out with primer *side_exoZ_out* and *Tc-out-Nter* (774 bp) and *side_exoP_out* and *Tc-out-Cter* (983 bp). The result is a LPU83 derivative with a deletion on the chromosome *exo* cluster.

The LPU83 mutant in the *exoB* gene was carried out by a simple crossing over. For this, a fragment of 295 bp of the *exoB* gene was amplified using *Phusion* polymerase and primers *exoB-Fw-int*/*exoB-Rv-int*. The product was cloned into the *SmaI* site of pK18mob, obtaining pK18mob-*exoB* (4088 bp). This vector was introduced by conjugation into *R. favelukesii* LPU83. Simple recombinants mutants were selected as Nm^*r*^. To corroborate the insertion, PCRs were carried out with primer *M13-Fw-40* and *exoB-Rv-comp* (1072 bp) and *M13-Rv-40* and *exoB-Fw-comp* (726 bp). The result is a LPU83 derivative with an insertion on the *exoB* gene.

The vector for the *exoB* complementation assay was constructed as follows. A fragment containing the full *exoB* gene was amplified (1372 bp) with *Phusion* polymerase and primers *exoB-Fw-comp*/*exoB-Rv-comp*. The fragment was cloned into the *SmaI* site of the broad host range vector pBBR1MCS-5. The orientation of the gene was evaluated to make sure that the Lac promoter that is present in the vector would express the *exoB* gene. The resulting vector was called pBBR1MCS5-*exoB* (6140 bp).

### Bioinformatics Approaches and Phylogenetic Analyses

For comparative genomics studies, the genes were searched for by means of BLASTp on the NCBI webpage. To examine genetic neighborhood, https://img.jgi.doe.gov/ was also used.

For the construction of the phylogenetic trees, the proteins were aligned with the module of Clustal implemented in MEGAX (Kumar et al., 2018). Prottest2.4 was used to determine the models of protein evolution (Abascal et al., 2005). The best model was LG+I+G for ExoB, LG+G+F for ExoH, LG+G for ExoV and LG+G for ExoY. Maximum likelihood (ML) trees were inferred under the selected model using PhyML v3.1 (Guindon and Gascuel, 2003). The robustness of the ML topologies was evaluated using a Shimodaira-Hasegawa-like test for branches implemented in PhyML v3.1 (values display in the nodes are multiplied by 100). We employed the best of NNIs and SPRs algorithms to search the tree topology and 100 random trees as initial trees. The accession numbers for the proteins selected for the phylogeny are display in each tree.

### Plant Assays

*Medicago sativa* (Alfalfa Sardi ten) seeds where surface-sterilized for 5 min in a 70% (v/v) ethanol solution, then washed with sterile distilled water. Subsequently, seeds were immersed in a solution of sodium hypochlorite (11 g l^–1^) for 15 min and washed six times with sterile distilled water. Seeds where placed on water agar (0.8% w/v) overnight. Afterward, seeds were placed on ethylene-oxide-sterilized plastic growth pouches containing 10 ml of Rolfe medium (Rolfe et al., 1980), with modifications (Barsch et al., 2006). Germination occurred in the pouches, and the roots developed through the hole in the paper. Seeds that did not germinate or did not grown through the hole were discarded. Three days post-germination, plants were inoculated with 10^6^ colony-forming units of each strain used in this work (at least 25 plants per strain). Plants were cultured in a growth chamber at 22°C with a 16-h photoperiod, watered with modified Rolfe medium and water. The CFU that each inocula contained were estimated by plate counts. Plant assays were repeated at least twice.

Plants were harvested 4 weeks after inoculation. Nodules were counted, weighted and conserved at 4°C. The shoot dry matter weight was measured. For nodule occupancy, nodules were sterilized and treated as previously described (Torres Tejerizo et al., 2010; Montiel et al., 2016). Briefly, the nodules that previously were weighted and counted, were surface-sterilized with H_2_O_2_ (30 volume) for 10 min, washed with sterile distilled water and then crushed in 200 ul of sterile isotonic solution followed by plating serial dilutions on TY with the corresponding antibiotics. 2–3 days after, CFU were counted.

For microscopy, nodules were embedded in 6% (w/v) agarose. Nodule sections of 60 μm were obtained using a Leica VT1000 S Vibratome. Fresh sections were then stained with Live/Dead BacLight bacterial Viability Kit (Thermo Fisher). The staining solutions were then removed and nodule sections were resuspended in PBS buffer. Images were acquired with a Leica TCS SP5 confocal microscope.

## Results and Discussion

### Localization and Gene Arrangement of Exopolysaccharide-Related Genes in *R. favelukesii* LPU83

During the symbiotic interaction between *E. meliloti* and alfalfa, different polysaccharides play relevant roles. Among them, EPS I (succinoglycan) is critical (Niehaus and Becker, 1998), but in some strains, the lack of EPS I may be overcome by EPS II (galactoglucan) or KPS (capsular polysaccharide, also referred as K antigen) (Pellock et al., 2000). As *R. favelukesii* LPU83 is able to infect alfalfa with some particular features (as mentioned in the introduction), the orthologs to the genes known to be involved in polysaccharides synthesis were searched for using BLAST. In addition to establishing a minimum of 35% identity as a cut-off to define orthologs (Rost, 1999), query coverage (>70%) and synteny were also considered in the analyses.

#### Capsular Polysaccharide

As mentioned in the Introduction, *Eme*AK631 only produces KPS (Putnoky et al., 1988; Putnoky et al., 1990; Reuhs et al., 1995) and, despite the lack of EPS I and EPS II, it is able to infect alfalfa (Putnoky et al., 1990; Pellock et al., 2000). Three clusters involved in KPS synthesis were described in *Eme*AK631 (Supplementary Figure 1): *rkp-1*, formed by *rkpABCDEFGHIJ* (Petrovics et al., 1993; Kiss et al., 1997), *rkp-2* which harbors *lpsL* and *rkpK* (Kereszt et al., 1998) and *rkp-3*, constituted by *rkpLMNOPQRSTZ* (Kiss et al., 2001). Later on, *rkpU* was found to be located upstream of *rkpA* in *Eme*AK631 and in *S. fredii* HH103 (Parada et al., 2006; Hidalgo et al., 2010). Recently, the parental strain of *Eme*AK631, *Eme*Rm41, was completely sequenced (Weidner et al., 2013). Surprisingly, in the annotation of the genome of *Eme*Rm41 a very large *rkpA* gene was detected, which comprises the *rkpABCDEF* genes from *Eme*AK631 (Supplementary Figure 1). A similar gene-fusion was observed in *Eme*1021 and *S. fredii* HH103 (Parada et al., 2006). By means of BLASTp, orthologs for the genes involved in KPS synthesis were searched for in LPU83. *rkpA* was not found as a large ORF in LPU83. We compared the genes of both, *Eme*AK631 and *Eme*2011. Some orthologs were found, but with low identity at the amino acid sequence (*ca.* 25–45%) and low coverage (Supplementary Tables 1, 2). In addition, none of the genes found were distributed in clusters.

For the *rkp-2* cluster, proteins with high similarity were found (for *lpsL*, LPU83_3231 and for *rkpK*, LPU83_3230). These genes showed *ca.* 70% identity and almost a full coverage in comparison to *Eme*2011 (Supplementary Figure 1). It is notable that in LPU83 *lpsL* is located downstream of *rkpK*, while in the *E. meliloti* strains the order of the genes is inverted, with *rkpK* situated downstream of *lpsL* (Supplementary Figure 1B).

For the genes that formed *rkp-3* cluster, again, differences were observed between *Eme*AK631 and *Eme*Rm41 (Supplementary Figure 1). Furthermore, it was observed that in *Eme*2011 the *rkp-3* cluster was not complete (Supplementary Figure 1), lacking *rkpLMNOPQ*. A similar lack of genes is observed in *Eme*1021 (not shown). In LPU83, only some orthologs were found, and they are not organized in clusters (Supplementary Table 3). The structure of KPS has been described in *Eme*AK631 (and also in other rhizobia) as repetitive units of hexose linked with 3-deoxy-D-*manno*-2-octulosonic acid (Kdo) or related 1-carboxy-2-keto-3-deoxy sugars (Reuhs et al., 1993; Reuhs et al., 1998; Fraysse et al., 2003). The KPS of *Eme*1021 is composed solely by Kdo and a phospholipid anchor (Pellock et al., 2000). *Eme*1021 has been shown to produce KPS, but it is biologically inefficient (Fraysse et al., 2005) and cannot support the symbiosis with alfalfa (Pellock et al., 2000).

The lack of orthologs in *Eme*2011 (and in *Eme*1021) to some of the genes involved in the biosynthesis of KPS (Supplementary Figure 1) could be related to the particular inefficient KPS described by Fraysse et al. (2005). Likewise, as LPU83 lacks almost all the orthologs for KPS synthesis, the probabilities of producing an efficient KPS should be very low.

#### Galactoglucan

*Ensifer meliloti* is able to synthetize, under specific environmental conditions or certain mutations, a galactoglucan called EPS II, which was described as a non-Calcofluor-binding exopolysaccharide that reacts with anthrone (Glazebrook and Walker, 1989). EPS II is composed of glucose and galactose residues in a 1:1 ratio. The proteins needed for its production are encoded in the *exp* gene cluster located in the pSymB (organized in five transcriptional units, see Supplementary Figure 2) (Becker et al., 2002). It has been demonstrated that strains producing EPS II, but neither EPS I nor KPS, are able to infect alfalfa, reinforcing a relevant role in the symbiosis (Pellock et al., 2000). Despite this infection being less efficient than that of strains expressing EPS I alone, EPS II is able to restore the nodule development and nitrogen fixation that is lacking in EPS I mutants (Glazebrook and Walker, 1989). For the production of EPS II, the transcriptional regulator ExpR must be expressed to activate the transcription of the genes in the *exp* cluster. ExpR is a homolog of the LuxR family regulators (Pellock et al., 2002). ExpR homologs were searched for in LPU83. Three hits were found, LPU83_4100 (65% identity and 97% coverage), LPU83_pLPU83b_0215 (41% identity and 47% coverage), and LPU83_pLPU83d_1676 (46% identity and 14% coverage), suggesting that some of them could play a similar role as that of ExpR in *E. meliloti*. Thus, the orthologs to the 21 genes that form the *exp* cluster of *Eme*2011 were searched in LPU83 (Supplementary Table 4). The results showed no hits for six genes (*expE8*, *expE5*, *expC*, *expA4*, *expA5*, and *expA6*) but for the remaining genes, some hits were found mainly dispersed among the chromosome and plasmids of LPU83. In most of the cases with low identity scores (*ca.* 30–40% of identity) and without synteny, with exception of the genes LPU83_pLPU83d_1427, LPU83_pLPU83d_1428, and LPU83_pLPU83d_1429 that are similar to *expD1*, *expD2*, and *expE1*, respectively. Altogether, and as will be shown below, the results suggest that no galactoglucan should be produced by LPU83.

Together with the already mentioned ExpR master regulator, *E. meliloti* possesses another master regulator of EPS biosynthesis, namely MucR representing a homolog of the RosR protein from *Agrobacterium tumefaciens* (Keller et al., 1995). Mutation of *mucR* increases EPS II production and reduces EPS I synthesis (Becker et al., 2002). It was also shown that MucR increases nod factor production (Mueller and Gonzalez, 2011). We found a homolog to MucR from *Eme*1021 (P55323.1) in LPU83, namely LPU83_1234 (81% identity and 99% coverage), which is actually annotated as *rosR* and is located on the chromosome. This suggests that EPS biosynthesis and nod factor production might be regulated by LPU83_1234, the MucR/RosR homolog.

#### Succinoglycan

**FIGURE 1:**
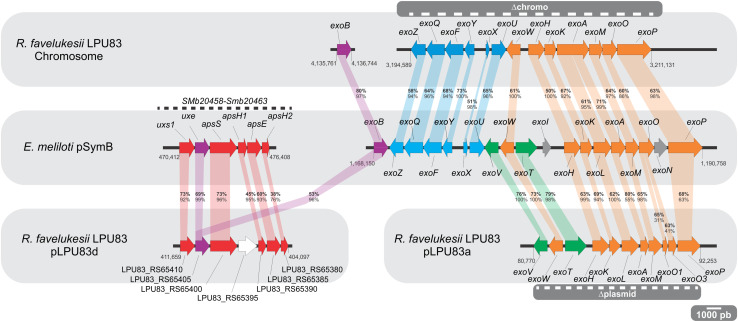
Genetic organization of the EPS I and APS genes. The *E. meliloti* pSymB figure (middle) shows the organization of the cluster of genes involved in APS (left) and EPS I (right) synthesis. The upper box shows the distribution of orthologs to EPS I production genes, distributed among *R. favelukesii* LPU83 chromosome. The bottom box shows the distribution of orthologs to APS (left) and EPS I (right) production genes, distributed among plasmids pLPU83d and pLPU83a, respectively. Numbers at the beginning and at the end of each cluster indicate the position of each gene. Orthologs are marked and connected with similar colors. Percentages in bold stand for amino acidic identity of the proteins. Percentages below bold-numbers stand for query coverage. All the comparisons were made using *E. meliloti* 2011 *exo* genes as query. The striped lines within gray boxes indicate the deletions made in the *exo* cluster of the chromosome and the plasmid.

Succinoglycan, also namely EPS I, is an acidic Calcofluor-binding exopolysaccharide that has been extensively studied and demonstrated as a key molecule for the proper symbiosis of *Eme*2011 with alfalfa (Leigh et al., 1985; Leigh et al., 1987; Niehaus and Becker, 1998; Jones et al., 2007; Marczak et al., 2017). EPS I is composed of repeating octasaccharidic subunits with glucose and galactose in a 7:1 ratio and different substituents (succinyl, acetyl, and pyruvyl) (Becker and Pühler, 1998; Fraysse et al., 2003). The synthesis of the EPS I is carried out by several proteins encoded in the gene cluster (*exoBZQFYXUVWTIHKLAMONP*) located in the pSymB (Figure 1). Mutants in *exoZ*, *exoX*, *exoI*, *exoK*, *exoO*, and *exoN* have been described as able to infect alfalfa [reviewed by Niehaus and Becker (1998)]. The genes involved in EPS I synthesis were searched for in the LPU83 genome using *E. meliloti* as query (Figure 1). Orthologs to *E. meliloti* genes were found distributed among a cluster in the chromosome (*exoZQFYXUWHKAMOP*), the smallest of the plasmids present in LPU83, pLPU83a (*exoVWTHKLAMO1O3P*) and elsewhere, but not clustered, in the chromosome (*exoB*, *exoI*–LPU83_RS49150 Id 33% Qc 92% and *exoN*–LPU83_RS55945 Id 65% Qc 92%) (Figure 1). In this case, the scores were higher in comparison to similar genes involved in EPS II or KPS biosynthesis. Remarkably, some genes are duplicated between the plasmid and chromosome clusters. *exoV* and *exoT* are only present in pLPU83a, while *exoZQFYXU* are only present in the chromosome of LPU83. *exoV* encodes for a glycosyltransferase that is necessary for the addition of the pyruvyl substituent (Glucksmann et al., 1993; Reuber and Walker, 1993). *exoT* encodes for a transmembrane protein needed for the polymerization and/or export of the EPS I (Becker et al., 1993; Glucksmann et al., 1993; Reuber and Walker, 1993). Both genes were early recognized as indispensable for the production of EPS I and for the infection of alfalfa by *E. meliloti* (Becker et al., 1993). Thus, the *exo* gene cluster present in pLPU83a could be essential for a proper EPS I production.

#### Arabinose-Containing Polysaccharide

It was recently shown that *Eme*2011 is also able to produce a Congo Red-binding extracellular polysaccharide (Schäper et al., 2017). This polysaccharide is an arabinose-containing polysaccharide (APS), whose production is enhanced when cyclic di-GMP levels are elevated. Through an over-expression of *pleD*, encoding a diguanylate cyclase, and *cuxR* encoding a c-di-GMP responsive transcriptional activator of the APS operon, Schäper et al. (2019) were able to study the regulation of genes involved in the synthesis of the APS operon: the operon *uxs1-uxe-apsS-apsH1-apsE-apsH2* (Figure 1). Strains that produce APS generate wrinkled red-colored macro-colonies on Congo Red containing medium (Schäper et al., 2019), but at this time there is no evidence of its role in symbiosis. A gene cluster of APS genes was found in pLPU83d, the largest plasmid of LPU83. Most of the APS-like genes showed identity values higher than 50%, with exception of *apsH1* and *apsH2* (Figure 1). Nevertheless, as it will be shown below, wrinkled macro-colonies were not observed in LPU83 nor in the mutants made in this work.

#### Cyclic β-Glucans (CG) and Mixed-Linkage β-Glucans (MLG)

Cyclic ß-glucans in *E. meliloti* consist of 17–25 glucose residues linked by ß-1,2 bonds [CGs bonds may be different in other rhizobia (Breedveld and Miller, 1994)]. CGs synthesis in *E. meliloti* is directed by the translated products of the genes *ndvA* and *ndvB*, and mutants in any of these genes are impaired in the symbiosis with alfalfa (Dylan et al., 1986; Dickstein et al., 1988; Stanfield et al., 1988; Jones et al., 2007). Homologs to *Eme*1021 NdvA (CAC47862.1) and NdvB (P20471.2) are present in strain LPU83, namely LPU83_4101 (CDM59736.1, 76% identity and 94% coverage when compared with NdvA) and LPU83_4179 (CDM59813.1 and 69% identity and 99% coverage when compared with NdvB). Despite such sequence similarity, *ndvA* and *ndvB* are not syntenic in *E. meliloti* and *R. favelukesii*. While in *Em*e1021 the *ndv* genes are separated by 3 Kbp, in *R. favelukesii* LPU83 the same genes are separated by more than 70 Kbp. Currently available data demonstrate that *ndvA* and *ndvB* are strongly transcribed at low extracellular pH in *R. favelukesii* LPU83 (Nilsson et al., 2020), a result that suggests a possible role of the CGs during the response of the bacteria to an increased concentration of extracellular hydrogen ions. Nonetheless, further biochemical analyses are necessary to investigate whether there is or not CGs production in *R. favelukesii* LPU83.

Under artificial increments of cyclic di-GMP levels it has been shown that *E. meliloti* 8530 is able to produce a different polysaccharide named mixed-linkage ß-glucans (MLGs). MLGs are linear (1→3) (1→4)-ß-D-glucans that are relevant for adhesion and colonization of alfalfa roots but not for nodule development (Pérez-Mendoza et al., 2015). Rhizobial colonies that produce MLGs bind both Calcofluor and Congo Red. The synthesis of MLGs in *E. meliloti* 8530 is mediated by BgsA (GT-2 protein, CAC48777.1) and BgsB (a membrane fusion protein, CAC48776.1). Homologs to *E. meliloti* BgsA and BgsB were found in *R. favelukesii* LPU83 (i.e., LPU83_pLPU83c_0651, CDM61213.1, 80% identity and 99% coverage; and LPU83_pLPU83c_0650, CDM61212.1, 68% identity and 100% coverage; respectively). Despite the synteny between the *bgs* genes in *E. meliloti* and *R. favelukesii*, we do not have yet evidence of MLGs production in strain LPU83.

The data presented suggests that LPU83 has the potential to produce EPS I and, under specific conditions, as increments of cyclic di-GMP levels, APS as stated by Schäper et al. (2017, 2019) or MLG as suggested by Pérez-Mendoza et al. (2015). Morphological observations of LPU83 show mucoid macro-colonies which predict the production of polysaccharides (Torres Tejerizo et al., 2016). The particular genetic organization that we found for the EPS I gene cluster raises some questions: which is the evolutionary relationship of the genes present in the plasmids? Are both genetic clusters essential for EPS synthesis? Is the EPS produced by LPU83 similar to the one produced by *E. meliloti*? Finally, how relevant is the EPS of LPU83 for the infection of alfalfa?

### Phylogenetic Analyses of EPS I Genes in *Rhizobium favelukesii* LPU83

The presence of two main clusters of *exo* genes in LPU83, one in the chromosome and another in plasmid pLPU83a point out that an event of horizontal gene transfer (HGT) could have occurred during the evolution of LPU83, where the strain acquired the *exo* genes from other bacteria. It is worth mentioning that previous work from our group has shown that pLPU83a is able to perform conjugative transfer (Torres Tejerizo et al., 2010), and the regulation of this phenomena depends on their genomic background and environmental conditions (Torres Tejerizo et al., 2014). Moreover, the plasmid gene cluster could be complementing the chromosomal genes to get a fully functional EPS I production.

**FIGURE 2:**
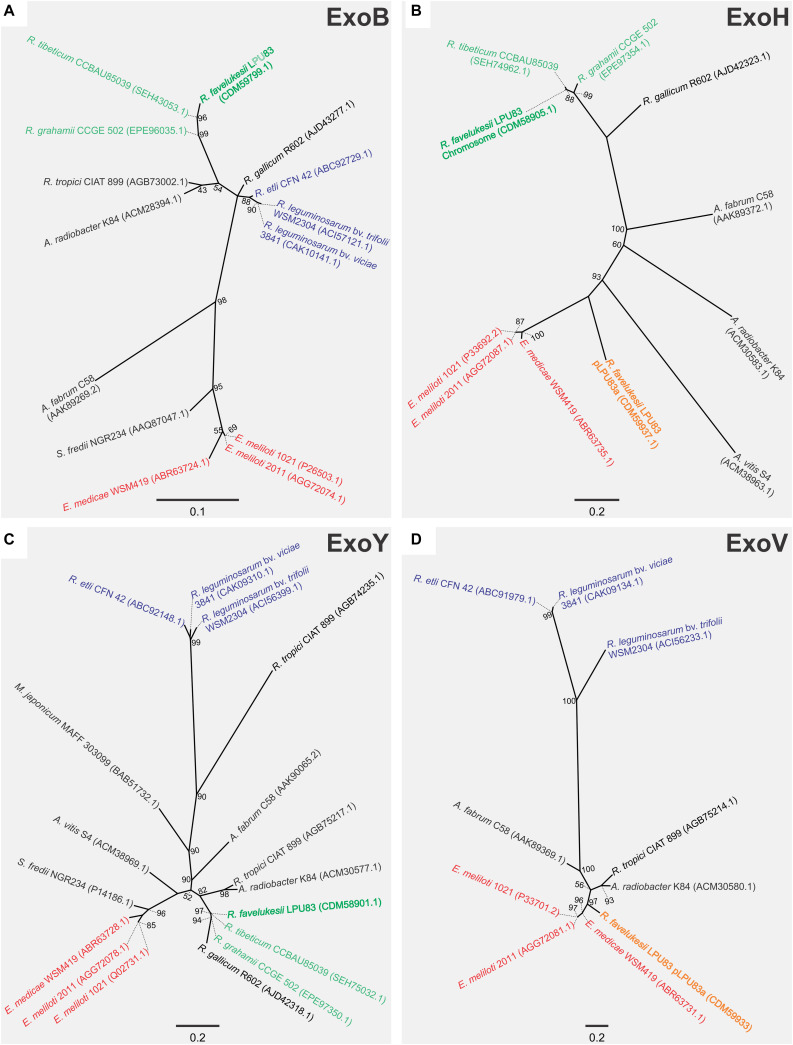
Phylogenetic analyses of EPS I genes of LPU83. Phylogenetic tree based on **(A)** ExoB (only in the chromosome of LPU83), **(B)** ExoH (in the chromosome of LPU83 and pLPU83a), **(C)** ExoY (only in the chromosome of LPU83), and **(D)** ExoV (only in the plasmid pLPU83a). Analyses were conducted by means of the Maximum Likelihood method. Support values (SH like × 100) greater than 50 are indicated at the nodes. Bars indicate substitution/site.

To understand if such event of HGT occurred, phylogenetic analyses were made employing four genes. One only present in the chromosomal gene cluster (*exoY*), one present only in the plasmid gene cluster (*exoV*), one present in both clusters (*exoH*) and the *bonafide exoB*, that was present in the chromosome but not in the *exo* gene cluster. For the phylogenetic inference, the translated products of the genes were used. The ExoB phylogenetic tree showed that it was related to *Rhizobium tibeticum* CCBAU85039 and *Rhizobium grahamii* CCGE 502 (Figure 2), strains with which it shares a close chromosomal relationship (Torres Tejerizo et al., 2016). In a similar way, ExoY (also harbored in the chromosome) clusters with the same strains. Meanwhile, ExoV, which is present in pLPU83a, groups with *E. meliloti* and *Ensifer medicae* strains. No orthologs to ExoV were found in *R. tibeticum* CCBAU85039 and *R. grahamii* CCGE 502. Finally, the phylogenetic tree of ExoH, locates the chromosomal-encoded copy near to *R. tibeticum* CCBAU85039 and *R. grahamii* CCGE 502 while the plasmid-encoded copy is closer to *E. meliloti* and *E. medicae*. These results support the hypothesis that the plasmid *exo* cluster arose from an HGT event, probably from an *Ensifer*-related strain. It was previously shown that the *exo* cluster present in the plasmid is flanked by two inverted repeats, which encode a Tn*3*-like transposase and recombinase (Castellani et al., 2019). These structures could be a reminiscence of an insertion/excision of foreign DNA.

Evidence of HGT events toward new-symbiotic strains has been shown previously, although the efficiency of the resulting symbioses may be low. Sullivan et al. (1995) and Sullivan and Ronson (1998) showed the lateral transfer of a symbiotic island from *Mesorhizobium loti* to non-symbiotic mesorhizobia allowing the evolution of the latter into symbiotic rhizobia. A similar event was observed in soils of the Brazilian Cerrados (Batista et al., 2007), where evidence of HGT from inoculated *Bradyrhizobium* strains to soil bacteria was detected. Recently, it was shown that the symbiotic plasmid of *Rhizobium etli* CFN42, or at least fragments of it, can be transferred to bean-endophytic bacteria, generating new strains with the ability to nodulate and fix nitrogen (Bañuelos-Vazquez et al., 2020). In our group, we showed that the gene cluster involved in the synthesis of the nodulation factors (NFs) of LPU83 has a structure similar to that of *E. meliloti*, with whom it also shares a close phylogenetic relationship (Del Papa et al., 2007; Torres Tejerizo et al., 2011). Thus, not only genes involved in NFs synthesis could have been recruited by HGT, but also a gene cluster involved in EPS biosynthesis, during the evolution of LPU83.

### The Exopolysaccharide Produced by *R. favelukesii* LPU83 Is Similar to the Succinoglycan of *E. meliloti*

**FIGURE 3:**
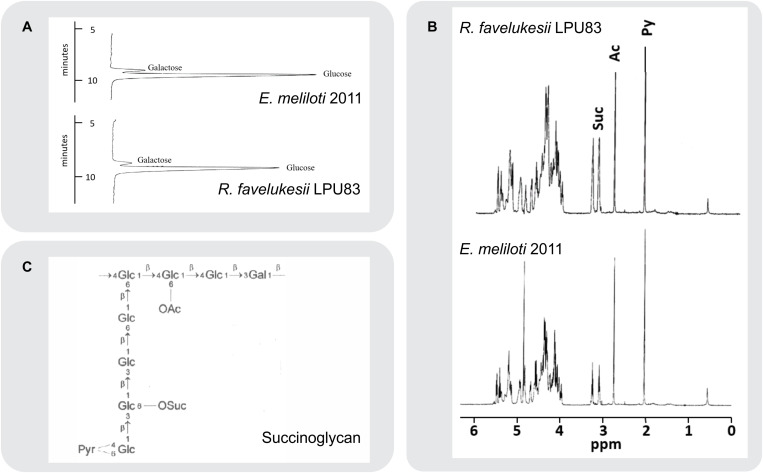
Composition and structural analyses of EPS. **(A)** Separation of monosaccharides obtained by acid hydrolysis of cetylpyridinium chloride (CPC) and ethanol precipitated culture supernatant of *E. meliloti* 2011 and *R. favelukesii* LPU83. The monosaccharides were separated by high-performance anion-exchange chromatography with pulsed amperometric detection on a Carbo Pac PA1 column. Only the relevant parts of the chromatograms are shown. **(B)** ^1^H-NMR spectra of isolated acidic exopolysaccharides (EPS) from *R. favelukesii* LPU83 and *E. meliloti* 2011. The singlets at 2.1 and 2.7 ppm represent the methyl protons from pyruvate and acetyl groups, respectively. The triplets at 3.1 and 3.25 ppm arise from the methylene protons of the succinyl group, while the complex region between 3.9 and 5.5 ppm represents signals from the ring protons of the carbohydrate constituents. **(C)** Structure of *E. meliloti* EPS I (succinoglycan). Diagram showing the octasaccharide repeating unit of succinoglycan (*OAc*, acyl group; *Pyr*, pyruvyl group; *OSuc*, succinyl group).

Several determinants, including EPS, play essential roles during the complex molecular crosstalk before and during the interaction between rhizobia and plants (Jones et al., 2007). Rhizobial EPS varies among strains and species, harboring different substitutions (Skorupska et al., 2006). Due to the relevant role of EPS during the symbiosis between rhizobia and *Medicago* spp. and the ability of LPU83 to nodulate *Medicago* spp. and other legumes (Wegener et al., 2001), the structure of the EPS produced by LPU83 was analyzed. EPS was precipitated from culture supernatants. Monosaccharide analysis of the hydrolyzed EPS of LPU83 indicated that the strain produces an EPS that consists of glucose and galactose in a ratio of 7 to 1, similar to EPS I of *Eme*2011 (Figure 3). The EPS of LPU83 was further analyzed by ^1^H-NMR and gas chromatography/mass spectrometry (GC/MS) studies. A ^1^H-NMR spectrum of EPS I isolated from *Eme*2011 was recorded as a control (Figure 3B). This spectrum was in accordance with data already published for this EPS I (Leigh et al., 1987; Keller et al., 1995). The proton resonances between 3.8 and 5.4 ppm were assigned to glycosyl components arising from ring protons, and the singlet resonances at 2.1 and 2.8 ppm were assigned to methyl protons of the 1-carboxyethylidene (pyruvate) and acetyl groups, respectively. The characteristic triplets at 3.1 and 3.25 ppm represent the methylene protons of the succinyl groups. The EPS isolated from LPU83 gave rise to virtually the same proton NMR spectrum, indicating that the main polysaccharide secreted by this strain is identical to succinoglycan from *Eme*2011.

At first glance, it may have been proposed that the different substitution/modifications of the EPS could be related to the failure in the nodule development and differentiation observed in *R. favelukesii* (Eardly et al., 1985; Del Papa et al., 1999; Wegener et al., 2001). Our results demonstrate that despite the different organization of EPS I genes, whose localization is not restricted to one cluster and that the evolution of both clusters seems to integrate genes from different rhizobial linages, the EPS 1 produced is identical to that of *E. meliloti*. The question that remains is whether these clusters are essential for this EPS production and plant infection or not.

### Genomic Regions Required for EPS Production of LPU83

**FIGURE 4:**
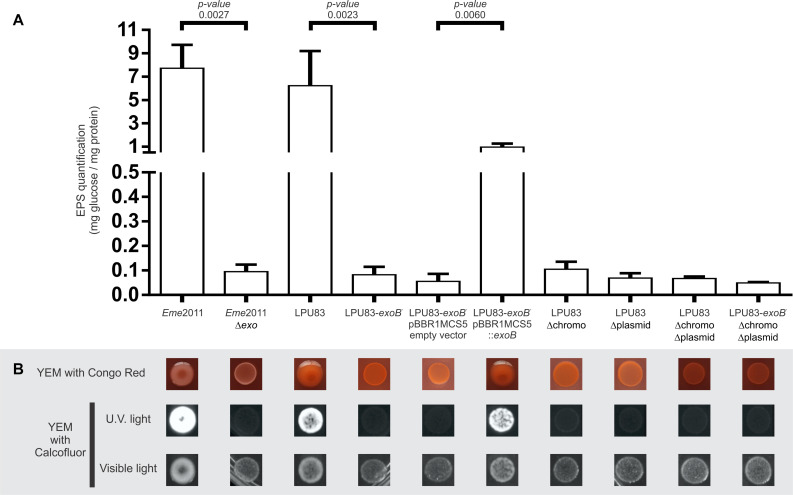
Characterization of EPS production of LPU83 mutants. **(A)** EPS quantification by the anthrone method. The EPS amount normalized per mg of total proteins is shown for each evaluated strain of *E. meliloti* and *R. favelukesii*. **(B)** Phenotypic analyses of the LPU83 mutants. EPS production was evaluated in YEM with Congo Red (first line) and in YEM with calcofluor under visible light and under UV light (lines below). The presence of EPS I is evidenced in the last condition due to its fluorescence. The statistical analysis was done by a *t*-test using three independent biological replicates. Representative pictures of at least three different experiments are shown.

We showed that LPU83 produces a succinoglycan identical to the one produced by *E. meliloti* and that the genes involved in the synthesis are distributed between the chromosome of LPU83 and plasmid pLPU83a. This particular organization leads to the question whether both gene clusters are relevant for the production of EPS I. To determine this, a genetic approach was used. The chromosome cluster was deleted by double crossing-over and inserting a resistance to Tc, generating strain LPU83 Δchromo (the region is indicated in Figure 1), as indicated in section “Materials and Methods”. The cluster located in pLPU83a was deleted in a similar way, but a Sp resistance was used instead, generating strain LPU83 Δplasmid (also indicated in Figure 1). ExoB has been recognized as a UDP-glucose 4-epimerase which provides the UDP-galactose in *E. meliloti* (Buendia et al., 1991). Mutants in *exoB* of *E. meliloti* fail to produce proper EPS I and EPS II due to the lack of galactose (Putnoky et al., 1990; Buendia et al., 1991; Reuhs et al., 1995). Due to the relevance of *exoB*, a mutant was also made. In this case, by single recombination through the integration of pK18mob-*exoB* plasmid (LPU83-*exoB*^–^). Afterward, the mutations were combined to generate a double deletion mutant (LPU83 Δchromo Δplasmid) and a triple mutant (LPU83-*exoB*^–^ Δchromo Δplasmid). Finally, LPU83-*exoB*^–^ was complemented in *trans* with a full copy of *exoB* (pBBR1MCS5-*exoB*). Wild type *Eme*2011 and *Eme*2011 Δ*exo* [which harbors a markerless deletion of the *exo* gene cluster from *exoP* to *exoZ* (Schäper et al., 2019)] were used as controls. EPS production was quantified with the anthrone method (Figure 4A) and EPS I was visualized due to its fluorescence in medium containing calcofluor (Figure 4B). The calcofluor assays demonstrated that only wild type strains (*Eme*2011 and LPU83) or the LPU83-*exoB*^–^ complemented in *trans* were able to produce EPS I. In agreement with this result, anthrone assays also showed EPS production by the same strains. The levels of EPS of the *exoB* mutant complemented were slightly lower than the wild type (Figure 4A), but the calcofluor fluorescence phenotype was clearly observed (Figure 4B).

Schäper et al. (2016, 2017) have shown that in *Eme*2011 EPS production is enhanced when cyclic di-GMP levels are elevated. Also, a new polysaccharide was described: an arabinose-containing polysaccharide (APS) (Schäper et al., 2019). The ectopical overexpression of *pleD* (diguanylate cyclase) and *cuxR* (c-di-GMP responsive transcriptional activator) leads to high di-GMP levels and, consequently, to an APS overproduction, generating wrinkled red-colored macro-colonies on medium containing Congo red (CR). Moreover, Pérez-Mendoza et al. (2015) showed that when cyclic di-GMP levels increase, MLGs can be produced. MLGs also bind Calcofluor and CR. The morphology of LPU83 and its mutants was evaluated in medium with CR, to determine binding of this dye. Neither LPU83 nor the mutants showed wrinkled macro-colonies nor red staining. Mutants of LPU83 did not show Calcofluor fluorescence (Figure 4). These results indicate that under our experimental conditions, APS and MLGs are not synthetized.

Exopolysaccharides production depends on many genes (Niehaus and Becker, 1998). In LPU83, the genes needed for the production of EPS I are distributed among different replicons. As shown above, both clusters and the *exoB* gene are needed for EPS production. In addition to the lack of orthologs of the genes needed for the synthesis of EPS II, the lack of glucose in the mutants suggest that galactoglucan is not produced in LPU83. The lack of CR staining suggests that also APS is not produced under our laboratory conditions. Altogether, LPU83 seems to be producing only EPS I, requiring both *exo* clusters and *exoB*.

### Symbiotic Phenotype of the LPU83 EPS Mutants

**FIGURE 5:**
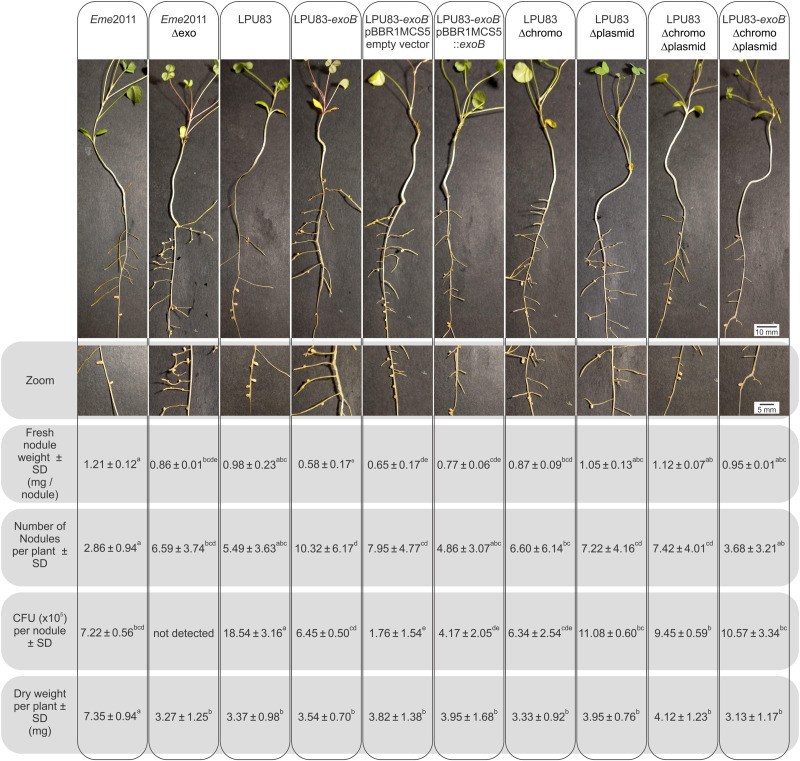
Analysis of the nodulation of LPU83 EPS I mutant strains. *M. sativa* plants infected by the indicated strains were harvested at 4 weeks post-inoculation. In the top panel, a representative plant was photographed. In second panel, zoom on the nodules of each plant was made. The number of nodules per plant were counted and weighted. Nodules were surfaced-sterilized, then crushed in sterile isotonic solution followed by plating on TY with the corresponding antibiotics and after 2 days CFU were counted. The shoot dry weight per plant was measured. Non-inoculated roots did not show nodules and the dry weight per plant was 3.98 ± 0.78^*b*^. Results were statistically analyzed by the ANOVA and least significant difference tests (Box et al., 1978). Values followed by different letters differed significantly with *p* < 0.05.

As mentioned earlier, KPS and EPS II may replace, at least partially, the lack of EPS I in the effective nodulation of alfalfa by *E. meliloti* (Pellock et al., 2000). But when only EPS I is produced, mutants in its biosynthesis fail to nodulate (Cheng and Walker, 1998; Niehaus and Becker, 1998; Mendis et al., 2016). Also in *Eme*2011, mutants in the genes involved in the sulfation of NFs are not able to nodulate (Roche et al., 1991; Schultze et al., 1995). LPU83 has the particular capacity to nodulate alfalfa even in absence of sulfated NFs (Torres Tejerizo et al., 2011). This peculiarity encouraged us to evaluate the nodulation of alfalfa using all the constructed strains. As control, we included *Eme*2011 and *Eme*2011 Δ*exo* strains. *Eme*2011 Δ*exo* is a deletion mutant that lacks the *exo* gene cluster from *exoP* to *exoZ*, but retains the *exoB* gene. *Eme*2011 Δ*exo* lacks the same genes as LPU83 Δchromo Δplasmid. Nodules induced by *Eme*2011 exhibited the typical cylindrical shape of alfalfa nodules with pink coloration. In contrast, *Eme*2011 Δ*exo* showed small white round nodules (Figure 5). Some of the nodules induced by wild-type LPU83 showed the typical cylindrical shape, however they were mostly white instead of pink. Similar to the nodules observed for the *Eme*2011 *exoB* mutant (Schäper et al., 2019), the nodules induced by LPU83-*exoB*^–^ and by LPU83-*exoB*^–^ (pBBR1MCS5 empty vector) were tiny and round, while the complemented mutant, LPU83-*exoB*^–^ (pBBR1MCS5-*exoB*), showed bigger nodules than LPU83-*exoB*^–^. The nodules induced by LPU83 Δplasmid were mainly round, but some of them were cylindrical. In contrast, the ones induced by LPU83 Δchromo were mainly cylindrical, but some were round. Unexpectedly, most of the nodules induced by the double and triple mutant were cylindrical. Nodules from all the derivatives of LPU83 were mostly white (Figure 5).

In the double and triple mutants, many nodules appeared with brown necrotic areas, while in the wild-type LPU83, these brown necrotic areas were observed in only a few nodules. These brown necrotic areas have been previously reported and related to plant defense symptoms, in nodules induced by EPS-deficient rhizobial mutants (Niehaus et al., 1993; Parniske et al., 1994; Fraysse et al., 2003; Berrabah et al., 2015). This led to the assumption that rhizobial EPS could act as suppressor of the plant defense response (Niehaus and Becker, 1998; Jones and Walker, 2008; Jones et al., 2008). This could cause the presence of the brown necrotic areas in nodules developed by LPU83 mutants, but it cannot explain their presence in the wild-type LPU83. One possibility is that the wild-type LPU83 triggers, at low levels, a plant defense mechanism. Studies with transcriptional fusions demonstrated that *exo* genes of *Eme*1021 were highly expressed in free-living cells but their expression decreased in later stages of symbiosis (Reuber et al., 1991). These observations were confirmed with recent high-throughput transcriptome studies (Capela et al., 2006; Roux et al., 2014). The expression of these genes might be fine-tuned by nodule signals, to allow a proper modulation of the plant defense. It cannot be disregarded that, despite the identical structure of EPS I of *Eme*2011 and LPU83, the spatial and temporal expression pattern of the *exo* genes could differ, causing a different response of the plant inside the nodules induced by LPU83.

Fresh nodules were counted, weighted and the number of CFU per nodule was evaluated (Figure 5). As expected, the comparison between *Eme*2011 and *Eme*2011 Δ*exo* showed statistical significant differences in fresh nodule numbers and weight. Among the LPU83 derivatives, wild-type LPU83, double and triple mutants and LPU83 Δplasmid showed similar fresh weight, while the LPU83-*exoB*^–^ showed lower fresh weight. LPU83-*exoB*^–^ and LPU83-*exoB*^–^ (pBBR1MCS5 empty vector) showed the highest numbers of nodules per plant, but harbored less bacteria inside. The complemented mutant, LPU83-*exoB*^–^ (pBBR1MCS5-*exoB*), generates similar numbers of nodules as LPU83, but less than the LPU83-*exoB*^–^ mutant. No CFU were detected in *Eme*2011 Δ*exo* nodules, indicating the lack of a proper infection. The absence of bacteria in mutants in EPS I production of *Eme*2011 was previously shown (Cheng and Walker, 1998; Niehaus and Becker, 1998). Nodules occupied by LPU83 showed the highest number of CFU per nodule. Unexpectedly, bacteria could be recovered from the nodules induced by all LPU83 mutants. LPU83 Δplasmid and the double and triple mutants showed CFU per nodule values similar to those of *Eme*2011. The estimation of nitrogen-fixing ability was evaluated by comparisons of the shoot-dry weights. As expected, *Eme*2011 showed nitrogen fixation, while *Eme*2011 Δ*exo* did not. LPU83 and its mutants showed a poor nitrogen fixation and the constructed mutations did not change the behavior of the wild-type strain (Figure 5).

**FIGURE 6:**
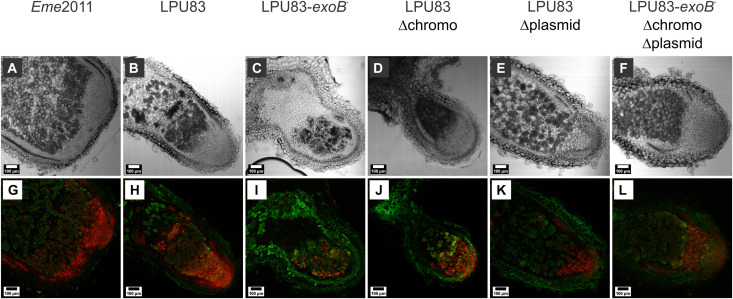
Morphology and occupancy of *M. sativa* nodules generated by EPS-I mutants of LPU83. Plants infected by *E. meliloti* 2011 and the indicated EPS I mutant strains of *R. favelukesii* LPU83 were harvested at 4 weeks post-inoculation. Nodule sections of 60 μm were obtained by means of a vibratome, and then stained with Syto 9 (green fluorescence that indicates living cells) and Propidium Iodide (red fluorescence that indicates cells with a damage in the membrane, i.e., dead). Light **(A–F)** and fluorescence **(G–L)** micrographs of full nodule slices were acquired with a confocal microscope. The photographs are representative of nodules of different plants experiments.

As the recovery of bacteria from the nodules induced by all LPU83 mutants was unexpected, nodule histology was evaluated by confocal microscopy. Nodules were cut and stained with Live-Dead BacLight. As a control, *Eme*2011 showed the typical tissue organization, where zones delimitation and living bacteria stained by SYTO9 were clearly visible (green fluorescence) and distributed in the nodule after the meristem (zone I) (Figures 6A,G). Nodules infected with LPU83 showed a similar pattern, but red-stained bacteria (indicative of dead bacteria) were observed. Moreover, less plant cells seem to be occupied by LPU83 than by *Eme*2011 (Figures 6B,H). The triple mutant, LPU83 Δplasmid and LPU83 Δchromo showed a similar pattern to the one observed in LPU83, but more cells seem to be alive. Also, more plant cells appear to be occupied by the mentioned mutants (Figures 6D–F,J–L). The LPU83-*exoB*^–^ strain showed smaller nodules, with an unclear definition of the zones and a large portion of the nodule was empty. Nevertheless, bacteria were observed inside these nodules but many of them were red-stained bacteria (Figures 6C,I). The size and shape of the nodules, the CFU per nodule obtained and the observed histology in nodules from LPU83-*exoB*^–^, show that the alteration in *exoB* severely impairs the nodulation of LPU83 but, contrary to *E. meliloti exoB* mutant (Schäper et al., 2019), LPU83-*exoB*^–^ is still able to infect. Remarkably, LPU83-*exoB*^–^ Δchromo Δplasmid, which lacks all *exo* genes, presents number of nodules, size, occupation and histology more similar to LPU83 than to the mutant LPU83-*exoB*^–^.

Overall, our results indicate that *R. favelukesii* LPU83 shows a lack of differentiation inside the nodules, which could explain the low nitrogen fixing rate. During the last years, nodule-specific cysteine-rich (NCRs) peptides have been intensively studied due to their role in the differentiation of the rhizobia inside the nodules (Van de Velde et al., 2010). Despite that NCRs show toxicity for the rhizobia *in vitro*, they are needed for the proper differentiation of the bacteria into bacteroids (Kondorosi et al., 2013; Pan and Wang, 2017). Furthermore, the membrane, the EPS and the LPS have been suggested as key components in the resistance to the NCRs (Montiel et al., 2017; Lima et al., 2020). It could be hypothesized that some other difference in the LPU83 cell envelope does not allow a correct perception of NCRs or that the expression of NCRs differs between the infection of *E. meliloti* and LPU83, resulting in non-proper symbiotic nodules in the case of infection by LPU83.

## Concluding Remarks

Legumes are of growing importance to reduce the global carbon dioxide footprint, since they do not need nitrogen fertilizers. The symbiosis between bacteria and leguminous plants has been therefore widely studied due its agronomical importance and also for providing the opportunity to understand the biochemical process involved in the communication between both partners (Jones et al., 2007). This communication includes several determinants from both organisms. One of them, the EPS, has been demonstrated as a key feature in different bacteria, with a role in recognition and signaling (Fraysse et al., 2003; Kawaharada et al., 2015). *E. meliloti* (especially the isogenic strains *Eme*1021 and *Eme* 2011) is considered as the model bacterium to study the symbiotic interaction between rhizobia and alfalfa. Nevertheless, *R. favelukesii* is a species that is also able to infect alfalfa, but its symbiosis is deficient. This failure seems to be related with the differentiation of the bacteria inside the nodules. Many determinants are involved in the differentiation, not only expressed in the late stages, but also during an early recognition. The synthesis of different EPS involves many genes, and their production could change according to different environmental conditions. The results presented here show that *R. favelukesii* produces an identical EPS I to the one produced by *E. meliloti*, whose synthesis is directed by genes located within a chromosomal cluster and a plasmid cluster. Both clusters have a different evolutionary origin and both of them are essential for the EPS I synthesis.

Remarkably, *R. favelukesii* does not need EPS I production to infect alfalfa. We observed that the mutants defective in EPS synthesis are able to generate nodules, with high titers of bacteria recovered from them. Noteworthy, a mutation affecting only *exoB* seems to be more severe than the lack of the whole *exo* genes. This could suggest that a punctual change in EPS I composition, as the lack of galactose, may be more harmful than the complete lack of it. In terms of specific determinants for the infection of alfalfa, *R. favelukesii* is a special species that differs from *E. meliloti*, since it is able to infect alfalfa, but with NFs that are somewhat different and with no EPS. Indeed, discerning the mechanism and determinants employed by *R. favelukesii* will lead us to a better understanding of the molecular pathway for a proper symbiosis and probably to alternative pathways or molecules for invasion that have yet to be described.

## Data Availability Statement

The original contributions presented in the study are included in the article/Supplementary Material, further inquiries can be directed to the corresponding author/s.

## Author Contributions

LC, ALu, JN, JPG, CW, MP, KN, and GTT did the experiments. AS, AP, ALa, SB, MP, KN, and GTT performed the data analysis and interpretation. LC, ALu, JN, and GTT wrote the manuscript. JPG, AS, AP, ALa, SB, MP, KN, and GTT manage the project and the fundings. All authors contributed to the discussion, provided comments on the manuscript, revised the manuscript, and have given approval to the final version of the manuscript.

## Conflict of Interest

The authors declare that the research was conducted in the absence of any commercial or financial relationships that could be construed as a potential conflict of interest.
