# Supplementary material for: Exopolysaccharide Characterization of Rhizobium favelukesii LPU83 and Its Role in the Symbiosis With Alfalfa
Source: Front Plant Sci. 2021 Feb 10;12:642576. doi: 10.3389/fpls.2021.642576 (PMC7902896; doi:10.3389/fpls.2021.642576)
Supplement: Supplementary file 1 [file Data_Sheet_1.pdf]

Supplemental Material

Exopolysaccharide characterization of *Rhizobium favelukesii* LPU83 and its role in the symbiosis with alfalfa

Lucas G. Castellani[1†], Abril Luchetti[1†], Juliet F. Nilsson[1], Julieta Pérez-Giménez[1], Caren Wegener[2], Andreas Schlüter[2], Alfred Pühler[2], Antonio Lagares[1], Susana Brom[3], Mariano Pistorio[1], Karsten Niehaus[2] and Gonzalo A. Torres Tejerizo[1]*.

[1]IBBM (Instituto de Biotecnología y Biología Molecular), CCT-La Plata, CONICET, Departamento de Ciencias Biológicas, Facultad de Ciencias Exactas, Universidad Nacional de La Plata, Calles 49 y 115 (1900). La Plata, Argentina
[2]CeBiTec, Bielefeld University, Bielefeld, Germany.
[3]Programa de Ingeniería Genómica, Centro de Ciencias Genómicas, Universidad Nacional Autónoma de México, Cuernavaca, Mor., México

Keywords: Rhizobia, exopolysaccharide, alfalfa, symbiosis, nitrogen fixation
Running title: Exopolysaccharides of *Rhizobium favelukesii*

[†] These authors have contributed equally to this work and share first autorship. Author order was determined alphabetically.
* Corresponding author: Gonzalo A. Torres Tejerizo
e-mail: gatt@biol.unlp.edu.ar

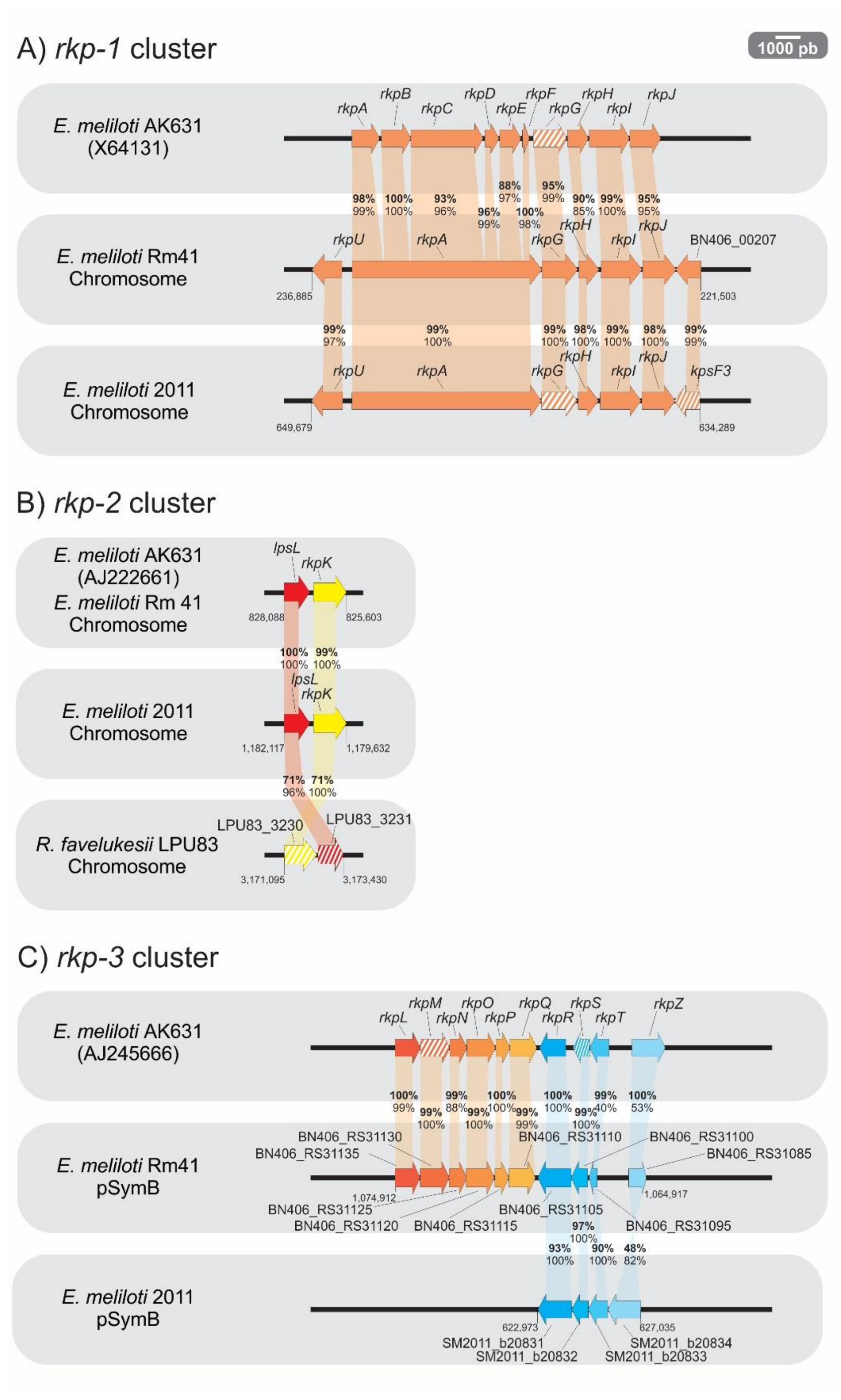

Fig. S1. Genetic organization of the Capsular Polysaccharide genes in *E. meliloti*. The figure shows the organization of the three clusters of genes involved in KPS synthesis. **A.** Distribution of orthologs to *rkp-1* cluster among *Eme*AK631, *Eme*Rm41 and *Eme*2011. **B.** Comparison of *rkp-2* cluster among *Eme*AK631, *Eme*2011 and *R. favelukesii* LPU83. **C.** Distribution of genes of *rkp-3* cluster among *Eme*AK631, *Eme*Rm41 and *Eme*2011. Numbers at the beginning and at the end of each cluster indicate the position in the replicon. Orthologs are marked and connected with similar colors. Percentages in bold stands for amino acidic identity of the proteins. Percentages below bold-numbers stands for query coverage. Striped genes stands for those genes that orthologs have been found in *R. favelukesii* LPU83. When genome projects are not available, accession numbers are indicated.

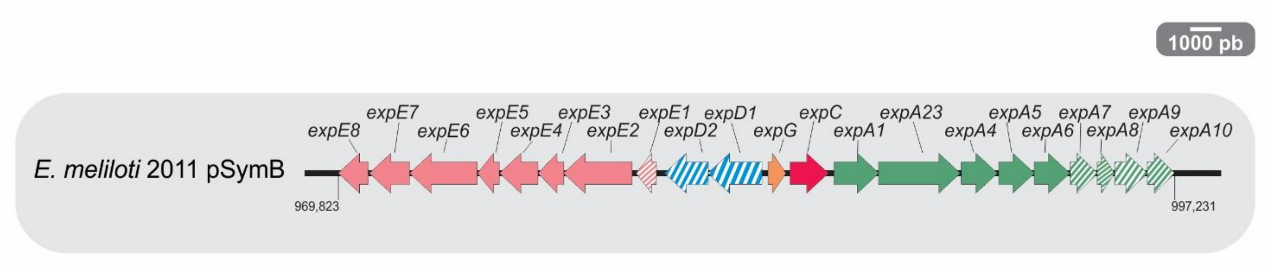

Fig. S2. Genetic organization of the galactoglucan (EPS II) of *E. meliloti* 2011. The figure shows the organization of the genes involved in EPS II biosynthesis. Numbers at the beginning and at the end of the cluster indicate the position in the replicon. Genes that are transcribe as operons are indicated with similar colors. Striped genes stands for those genes that orthologs have been found in *R. favelukesii* LPU83.

Table S1. BLASTp hits of *rkp-1* cluster of Capsular Polysaccharide genes of *E. meliloti* AK631 in LPU83

**Query: *E. meliloti* AK631 proteins**				**LPU83 Proteins**				
						**Position**		
Gene	Protein ID	Identity (%)	Query Coverage (%)	Locus Tag	Protein ID	Replicon / Contig [a]	From	To
rkpA	CAA45483	28	75	LPU83_1401	CDM57074	HG916852	1385340	1386602
		27	73	LPU83_pLPU83b_0505	CDM60487	CBYB010000047	9996	8788
rkpB	CAA45484	No hit						
rkpC	CAA45485	44	3	LPU83_pLPU83c_0420	CDM60982	HG916854	391746	392750
		41	3	LPU83_2442	CDM58097	HG916852	2380887	2381867
rkpD	CAA45486	41	64	LPU83_pLPU83c_0546	CDM61108	HG916854	512413	511412
		40	65	LPU83_2442	CDM58097	HG916852	2380887	2381867
		31	94	LPU83_1121	CDM56795	HG916852	1106617	1107612
rkpE	CAA45487	42	25	LPU83_pLPU83d_1332	CDM62702	HG916855	1341450	1340620
		31	28	LPU83_1399	CDM57072	HG916852	1383981	1384718
rkpF	CAA45488	No hit						
rkpG	CAA45489	39	72	LPU83_pLPU83d_0028	CDM61399	HG916855	27218	26073
		32	75	LPU83_2854	CDM58505	HG916852	2799901	2798714
rkpH	CAA45490	28	66	LPU83_1822	CDM57486	HG916852	1767878	1767102
		27	61	LPU83_3024	CDM58675	HG916852	2974708	2975484
rkpI	CAA45491	34	9	LPU83_0054	CDM55745	HG916852	55219	55713
rkpJ	CAA45492	41	9	LPU83_pLPU83d_1678	CDM63048	HG916855	1664794	1663703
		41	8	LPU83_pLPU83a_0096	CDM59937	HG916853	84821	85987

[a]HG916852: Chromosome; HG916853: pLPU83a; HG916854: pLPU83c; HG916855: pLPU83d; CBYB010000047: contig of pLPU83b (symbiotic plasmid)

Table S2. BLASTp hits of *rkp-1* cluster of Capsular Polysaccharide genes of *E. meliloti* 2011 in LPU83

**Query: *E. meliloti* 2011 proteins**				**LPU83 Proteins**				
						Position		
Gene	Protein ID	Identity (%)	Query Coverage (%)	Locus Tag	Protein ID	Replicon / Contig [a]	From	To
rpkU	AGG73158	No hit						
rkpA	AGG73157	37	9	LPU83_2442	CDM58097	HG916852	2380887	2381867
		30	12	LPU83_1121	CDM56795	HG916852	1106617	1107612
rkpG	AGG73156	39	71	LPU83_pLPU83d_0028	CDM61399	HG916855	27218	26073
		32	76	LPU83_2854	CDM58505	HG916852	2798714	2799901
rkpH	AGG73155	36	67	LPU83_pLPU83d_0842	CDM62212	HG916855	850711	849965
		31	71	LPU83_0423	CDM56106	HG916852	410004	409240
rkpI	AGG73154	No hit						
rkpJ	AGG73153	34	13	LPU83_pLPU83d_1678	CDM63048	HG916855	1664794	1663703
kpsF3	AGG73152	49	95	LPU83_3551	CDM59195	HG916852	3500969	3499974
		26	64	LPU83_pLPU83b_0254	CDM60245	CBYB010000026	6620	8446

[a]HG916852: Chromosome; HG916853: pLPU83a; HG916854: pLPU83c; HG916855: pLPU83d; CBYB010000026: contig of pLPU83b (symbiotic plasmid)

Table S3. BLASTp hits of *rkp-3* cluster of Capsular Polysaccharide genes of *E. meliloti* AK631 in LPU83

**Query: *E. meliloti* AK631 proteins**				**LPU83 Proteins**				
						Position		
Gene	Protein ID	Identity (%)	Query Coverage (%)	Locus Tag	Protein ID	Replicon / Contig [a]	From	To
rkpL	CAB62150	33	83	LPU83_pLPU83d_1458	CDM62828	HG916855	1470461	1468536
rkpM	CAB62151	33	99	LPU83_pLPU83d_0236	CDM61607	HG916855	261312	262490
		35	94	LPU83_pLPU83d_0233	CDM61604	HG916855	258531	259637
rkpN	CAB62152	No hit						
rkpO	CAB62153	36	11	LPU83_pLPU83b_0567	CDM60547	CBYB010000056	248	1222
rkpP	CAB62154	31	47	LPU83_2720	CDM58372	HG916852	2655893	2656372
		28	60	LPU83_1074	CDM56750	HG916852	1062217	1061603
rkpQ	CAB62155	No hit						
rkpR	CAB62156	No hit						
rkpS	CAB62157	35	83	LPU83_pLPU83d_1091	CDM62461	HG916855	1077195	1077923
		29	92	LPU83_pLPU83d_0289	CDM61660	HG916855	329946	328423
rkpT	CAB62158	28	28	LPU83_0485	CDM56168	HG916852	475173	474028
		62	6	LPU83_pLPU83c_0797	CDM61359	HG916854	751841	752410
rkpZ	CAB62159	48	5	LPU83_pLPU83b_0279	CDM60269	CBYB010000030	5521	7311

[a]HG916852: Chromosome; HG916853: pLPU83a; HG916854: pLPU83c; HG916855: pLPU83d; CBYB010000030 and CBYB010000056: contigs of pLPU83b (symbiotic plasmid)

Table S4. BLASTp hits of EPS II genes of *E. meliloti* 2011 in LPU83

**Query *E. meliloti* 2011 proteins**			**LPU83 Proteins**						
							Position		
Gene	Locus Tag	Protein ID	Identity (%)	Query Coverage (%)	Locus Tag	Protein ID	Replicon / Contig [a]	From	To
expE8	SM2011_b21307	AGG71891	No Hit						
expE7	SM2011_b21308	AGG71892	30	31	LPU83_pLPU83d_1089	CDM62459	HG916855	1075119	1076369
			26	39	LPU83_2876	CDM58527	HG916852	2826202	2825189
expE6	SM2011_b21309	AGG71893	35	6	LPU83_pLPU83d_1270	CDM62640	HG916855	1279333	1276391
expE5	SM2011_b21310	AGG71894	No Hit						
expE4	SM2011_b21311	AGG71895	30	39	LPU83_pLPU83d_0326	CDM61697	HG916855	368053	370320
			27	55	LPU83_pLPU83d_1124	CDM62494	HG916855	1114835	1115923
expE3	SM2011_b21312	AGG71896	33	44	LPU83_pLPU83b_0573	CDM60553	CBYB010000057	3165	2443
			29	43	LPU83_3772	CDM59410	HG916852	3747301	3748047
expE2	SM2011_b21313	AGG71897	29	22	LPU83_pLPU83d_0223	CDM61594	HG916855	245184	246143
expE1	SM2011_b21314	AGG71898	31	86	LPU83_pLPU83d_1429	CDM62799	HG916855	1439682	1441118
			36	92	LPU83_1147	CDM56821	HG916852	1131116	1142074

expD2	SM2011_b21315	AGG71899	31	90	LPU83_pLPU83d_1428	CDM62798	HG916855	1438316	1439623
			34	31	LPU83_pLPU83d_0411	CDM61782	HG916855	455414	455869
expD1	SM2011_b21316	AGG71900	39	89	LPU83_pLPU83d_1427	CDM62797	HG916855	1436568	1438319
			28	91	LPU83_1150	CDM56824	HG916852	1144875	1147037
expG	SM2011_b21317	AGG71901	39	61	LPU83_1462	CDM57135	HG916852	1450683	1451198
expC	SM2011_b21318	AGG71902	No Hit						
expA1	SM2011_b21319	AGG71903	35	8	LPU83_pLPU83d_1232	CDM62602	HG916855	1230902	1230069
expA23	SM2011_b21320	AGG71904	26	15	LPU83_pLPU83d_1124	CDM62494	HG916855	1114835	1115923
			37	10	LPU83_pLPU83a_0093	CDM59934	HG916853	82781	81822
expA4	SM2011_b21321	AGG71905	No Hit						
expA5	SM2011_b21322	AGG71906	No Hit						
expA6	SM2011_b21323	AGG71907	No Hit						
expA7	SM2011_b21324	AGG71908	62	93	LPU83_pLPU83d_0157	CDM61528	HG916855	164605	163721
			59	64	LPU83_3553	CDM59197	HG916852	3503572	3502685

expA8	SM2011_b21325	AGG71909	49	97	LPU83_pLPU83d_0156	CDM61527	HG916855	163724	163164
			42	90	LPU83_0092	CDM55778	HG916852	101986	102540
expA9	SM2011_b21326	AGG71910	54	91	LPU83_pLPU83d_0159	CDM61530	HG916855	166544	165495
			25	93	LPU83_3231	CDM58881	HG916852	3172258	3173430
expA10	SM2011_b21327	AGG71911	39	97	LPU83_pLPU83d_0158	CDM61529	HG916855	165498	164602
			41	9	LPU83_2676	CDM58328	HG916852	2608339	2609229

[a]HG916852: Chromosome; HG916853: pLPU83a; HG916854: pLPU83c; HG916855: pLPU83d; CBYB010000057: contig of pLPU83b (symbiotic plasmid)

Table S5. Bacterial strains and plasmids used in this study

**Name**	**Description**	**Resistance**	**Reference**
**Strains**			
Ensifer meliloti 2011	Wild type strain. Nod$^+$ Fix$^+$ in alfalfa	Sm	(Casse et al., 1979)
Ensifer meliloti 2011 Δ*exo*	Derivative of *Eme*2011 with markerless deletion of the *exo* gene cluster from *exoP* to *exoZ*		(Schaper et al., 2019)
R. favelukesii LPU83	Wild type strain	Sm	(Del Papa et al., 1999)
R. favelukesii LPU83-*exoB*$^-$	Derivative of LPU83, *exoB*::pK18mob	Sm Nm	This work
R. favelukesii LPU83-*exoB*$^-$ (pBBR1MCS5 empty vector)	Derivative of LPU83-*exoB*$^-$, carrying pBBR1MCS5	Sm Nm Gm	This work
R. favelukesii LPU83 *exoB*$^-$ (pBBR1MCS5-*exoB*)	Derivative of LPU83-*exoB*$^-$, carrying pBBR1MCS5-*exoB*	Sm Nm Gm	This work
R. favelukesii LPU83 Δchromo	Derivative of LPU83 with a deletion of the chromosomal *exo* gene cluster from *exoZ* to *exoP*	Sm Tc	This work
R. favelukesii LPU83 Δplasmid	Derivative of LPU83 with a deletion of the plasmid *exo* gene cluster from *exoV* to *exoP*	Sm Sp	This work
R. favelukesii LPU83 Δchromo Δplasmid	Derivative *R. favelukesii* LPU83 Δchromo which receive plasmid pLPU83a from *R. favelukesii* LPU83 Δplasmid	Sm Tc Sp	This work
R. favelukesii LPU83-*exoB*$^-$ Δchromo Δplasmid	Derivative of *R. favelukesii* LPU83 Δchromo Δplasmid, *exoB*::pK18mob	Sm Nm Tc Sp	This work
E. coli DH5α	*supE44 ΔlacU169 ϕ80dlacZΔM15 hsdR171 recA1 endA1 gyrA96 thi-1 relA1*		Bethesda Res. Lab.
E. coli S17–1	*E. coli* 294 RP4-2-Tc::Mu-Km::Tn*7* integrated into the chromosome		(Simon et al., 1983)
**Plasmids**			

pRK2013	Helper plasmid in triparental matings	Km	(Figurski and Helinski, 1979)
pCR 2.1-TOPO	High copy number cloning vector	Amp Km	Invitrogen
pHP45-Sp	Vector carrying a DNA cassette for Sp^R flanked by transcription and translation terminators	Amp Sp	(Fellay et al., 1987)
pHP45-Tc	Vector carrying a DNA cassette for Tc^R flanked by transcription and translation terminators	Amp Tc	(Fellay et al., 1987)
pTOPO-*exoV*	pCR 2.1-TOPO carrying a 414 bp fragment of the *exoV* gene from pLPU83a	Amp Km	This work
pTOPO-*exoV*-Sp	Derivative of pTOPO-*exoV*, carrying ΩSp-*SmaI* fragment from pHP45-Sp in the *EcoRV* site	Amp Km Sp	This work
pTOPO-*exoPp*	pCR 2.1-TOPO carrying a 382 bp fragment of the *exoP* gene from pLPU83a	Amp Km	This work
pK18mobSacB	Cloning vector, mobilizable	Km	(Schäfer et al., 1994)
pK18mobSacB-*exoPp*	Derivative of pK18mobSacB, carrying a 400 bp *EcoRI*-fragment of the *exoP* gene from pTOPO-*exoPp*	Km	This work
pK18mobSacB-*exoV*-Sp-*exoPp*	Derivative of pK18mobSacB-*exoPp*, carrying a *SpeI*/*XhoI* fragment (2576 pb) of TOPO-*exoV*-Sp in the *NheI*/*SalI* site of pK18mobSacB-*exoPp*	Km Sp	This work
pK18mob	High copy number cloning vector	Km	(Schäfer et al., 1994)
pK18mob-*exoPc*	Derivative of pK18mobSacB, carrying a 210 bp fragment of the *exoP* gene from chromosome of LPU83	Km	This work
pK18mobSacB-*exoPc*	Derivative of pK18mobSacB, carrying a 216 bp *BamHI*/*HindIII* fragment of pK18mob-*exoPc*	Km	This work
pK18mob-*exoZ*	Derivative of pK18mobSacB, carrying a 185 bp fragment of the *exoZ* gene from chromosome of LPU83	Km	This work
pK18mobSacB-*exoZ*-*exoPc*	Derivative of pK18mobSacB-*exoPc*, carrying a 193 bp *EcoRI*/*SmaI* fragment of pK18mob-*exoZ* in the *EcoRI*/*SmaI* sites of pK18mobSacB-*exoPc*	Km	This work
pK18mobSacB-*exoZ*-Tc:*exoPc*	Derivative of pK18mobSacB-*exoZ*-*exoPc*, carrying ΩTc-*SmaI* fragment from pHP45-Sp in the *SmaI* site	Km Tc	This work

pK18mob-*exoB*	Derivative of pK18mob carrying a 295 bp fragment of the *exoB in the SmaI* site	Km	This work
pBBR1MCS5	Broad-host-range cloning vector, mobilizable	Gm	(Kovach et al., 1995)
pBBR1MCS5-*exoB*	Derivative of pBBR1MCS5 carrying a complete copy (1372 pb) of *exoB in the SmaI* site	Gm	This work

Nm^r, Sm^r, Tc^r, Gm^r, Km^r, Amp^r and Sp^r= neomycin, streptomycin, tetracycline, gentamicin, kanamycin, ampicillin, and spectinomycin resistance, respectively.

Table S6. Oligonucleotides used in this study

Name	Sequence	Reference
M13-rv40	CAGGAAACAGCTATGAC	Universal primer
M13-fw40	GTTTTCCCAGTCACGAC	Universal primer
exoB-Fw-comp	CGCGAGAACAATCCGAAG	This work
exoB-Rv-comp	GCCTCGCCTGATGGAAC	This work
exoB-Fw-int	GGATCGGGCTCGTCTTG	This work
exoB-Rv-int	CGCCTGCTCGATGATGT	This work
side_exoZ_Fw_cro83_Eco	AAAAAAGAATTCCCGCTTGCCATCATCC T	This work
side_exoZ_Rv_cro83_Sma	AAAAAACCCGGGGCCGATTTGCCTTCGT C	This work
side_exoP_Fw_cro83_Ba m	AAAAAAGGATCCCGGCGTGATCCTCAA CA	This work
side_exoP_Rv_cro83_Hin	AAAAAAAAGCTTTCCACAGCGGCAAAC TC	This work
Tc-out-Nter	GGCGAGATCACCAAGGTA	This work
Tc-out-Cter	CTCGACCTGAATGGAAGC	This work
side_exoZ_out	TGCCAGCAGGAACAACCT	This work
Sm-Sp	CGGTGGATGACCTTTTGAAT	(Quelas et al., 2010)
L_exoV-BamHI-del-LEFT	AAAAGGATCCCATGGCGCATTGGAAAA	This work
L_exoV-XbaI-del-RIGHT	AAAATCTAGATGAGCGCGGATCAGGT	This work
L_exoV-out-LEFT	GCTGGAGGCAACGACCT	This work
L_exoP-BamHI-del-LEFT	AAAAGGATCCCTCGTTCTTGGCGGTCTC	This work
L_exoP-XbaI-del-RIGHT	AAAATCTAGATCCGTTCCTCGCTTTCC	This work
L_exoP-out-RIGHT	GGAAATCGCCCCAAAGA	This work

References

Casse, F., Bouche, C., Julliot, J.S., Michel, M., and Dénarié, J. (1979). Identification and Characterization of Large Plasmids in Rhizobium meliloti using Agarose Gel Electrophoresis. *Microbiology* 113**,** 229-242. doi: 10.1099/00221287-113-2-229

Del Papa, M.F., Balagué, L.J., Sowinski, S.C., Wegener, C., Segundo, E., Abarca, F.M., et al. (1999). Isolation and characterization of alfalfa-nodulating rhizobia present in acidic soils of central argentina and uruguay. *Appl Environ Microbiol* 65**,** 1420-1427. doi: 10.1128/AEM.65.4.1420-1427.1999

Fellay, R., Frey, J., and Krisch, H. (1987). Interposon mutagenesis of soil and water bacteria: a family of DNA fragments designed for in vitro insertional mutagenesis of gram-negative bacteria. *Gene* 52**,** 147-154. doi: 10.1016/0378-1119(87)90041-2

Figurski, D.H., and Helinski, D.R. (1979). Replication of an origin-containing derivative of plasmid RK2 dependent on a plasmid function provided in trans. *Proc Natl Acad Sci U S A* 76**,** 1648-1652. doi: 10.1073/pnas.76.4.1648

Kovach, M.E., Elzer, P.H., Hill, D.S., Robertson, G.T., Farris, M.A., Roop, R.M., 2nd, et al. (1995). Four new derivatives of the broad-host-range cloning vector pBBR1MCS, carrying different antibiotic-resistance cassettes. *Gene* 166**,** 175-176. doi: 10.1016/0378-1119(95)00584-1

Quelas, J.I., Mongiardini, E.J., Casabuono, A., Lopez-Garcia, S.L., Althabegoiti, M.J., Covelli, J.M., et al. (2010). Lack of galactose or galacturonic acid in Bradyrhizobium japonicum USDA 110 exopolysaccharide leads to different symbiotic responses in soybean. *Mol Plant Microbe Interact* 23**,** 1592-1604. doi: 10.1094/MPMI-05-10-0122

Schäfer, A., Tauch, A., Jäger, W., Kalinowski, J., Thierbach, G., and Pühler, A. (1994). Small mobilizable multi-purpose cloning vectors derived from the *Escherichia coli* plasmids pK18 and pK19: selection of defined deletions in the chromosome of *Corynebacterium glutamicum*. *Gene* 145**,** 69-73. doi: 10.1016/0378-1119(94)90324-7

Schaper, S., Wendt, H., Bamberger, J., Sieber, V., Schmid, J., and Becker, A. (2019). A Bifunctional UDP-Sugar 4-Epimerase Supports Biosynthesis of Multiple Cell Surface Polysaccharides in *Sinorhizobium meliloti*. *J Bacteriol* 201. doi: 10.1128/JB.00801-18

Simon, R., Priefer, U., and Pühler, A. (1983). A broad host range mobilization system for in vivo genetic engineering: Transposon mutagenesis in gram negative bacteria. *Bio/Technology* 1**,** 784-791. doi: 10.1128/jb.177.1.52-58.1995
